# New Nanofiber Composition for Multiscale Bubble Capture and Separation

**DOI:** 10.1021/acsomega.2c04426

**Published:** 2022-10-25

**Authors:** Araz Sheibani Aghdam, Farzad Rokhsar Talabazar, Mohammad Jafarpour, Ali Koşar, Fevzi Çakmak Cebeci, Morteza Ghorbani

**Affiliations:** † Sabanci University Nanotechnology Research and Application Center, 34956 Tuzla, Istanbul, Turkey; ‡ Faculty of Engineering and Natural Sciences, Sabanci University, 34956 Tuzla, Istanbul, Turkey; § Center of Excellence for Functional Surfaces and Interfaces for Nano-Diagnostics (EFSUN), Sabanci University, Orhanli, 34956 Tuzla, Istanbul, Turkey

## Abstract

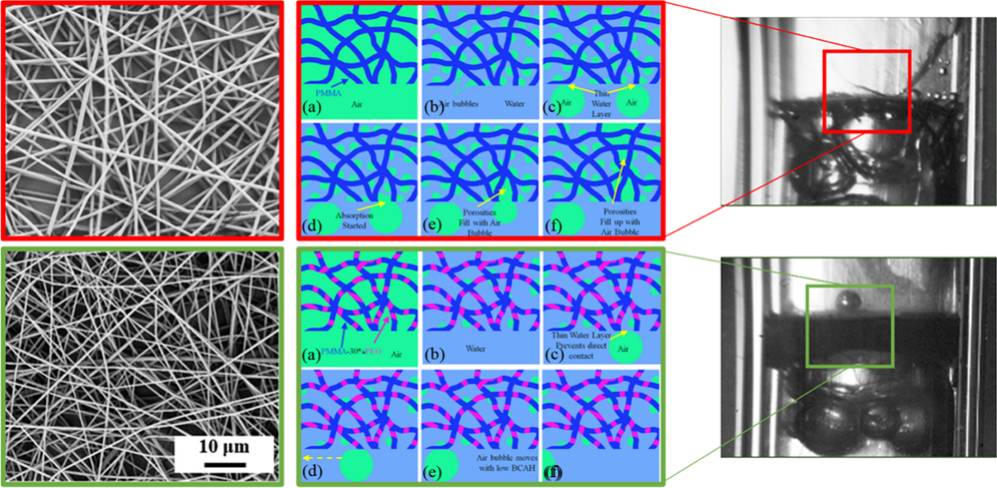

Bubble dynamics inside a liquid medium and its interactions with hydrophobic and hydrophilic surfaces are crucial for many industrial processes. Electrospinning of polymers has emerged as a promising fabrication technique capable of producing a wide variety of hydrophobic and hydrophilic polymer nanofibers and membranes at a low cost. Thus, knowledge about the bubble interactions on electrospun hydrophobic and hydrophilic nanofibers can be utilized for capturing; separating; and transporting macro-, micro-, and nanobubbles. In this study, poly(methyl methacrylate) (PMMA) and PMMA–poly(ethylene glycol) (PEG) electrospun nanofibers were fabricated to investigate gas bubble interactions with submerged nanofiber mats. To improve their durability, the nanofibers were reinforced with a plastic mesh. The ultimate tensile strengths of PMMA and PMMA-30%PEG nanofibers were measured as 0.35 and 0.30 MPa, respectively. With the use of reinforcement mesh, the mechanical properties of final membranes could be improved by a factor of 70. The gas permeability of the electrospun and reinforced nanofibers was also studied using the high-speed visualization technique and a homemade setup to investigate the effect of electrospun nanofibers on the bubble coalescence and size in addition to the frequency of released bubbles from the nanofiber mat. The diffusion rate of air bubbles in hydrophobic PMMA electrospun nanofibers was measured as 10 L/s for each square meter of the nanofiber. However, the PMMA-30%PEG mat was able to restrict the diffusion of gas bubbles through its pores owing to the van der Waals force between the water molecules and nanofiber surface as well as the high stability of the thin water layer. It has been shown that the hydrophobic electrospun nanofibers can capture and coalesce the rising gas bubbles and release them with predictable size and frequency. Consequently, the diameter of bubbles introduced to the hydrophobic PMMA membrane ranged between 2 and 25 mm, whereas the diameter of bubbles released from the hydrophobic electrospun nanofibers was measured as 8 ± 1 mm. The proposed mechanism and fabricated electrospun nanofibers can enhance the efficiency of various systems such as heat exchangers, liquid–gas separation filters, and direct air capture (DAC) systems.

## Introduction

Recent developments in surface engineering and synthesis of hydrophobic and hydrophilic surfaces have enabled their applications in a variety of fields, including biotechnology,^1^ self-cleaning technology,^2^ and the petroleum industry.^3^ Meanwhile, bubble dynamics in a liquid medium and its interactions with hydrophobic and hydrophilic surfaces are critical in many industrial applications such as cavitation erosion,^4^ drag reduction,^5^ froth flotation,^6^ lab-on-chip devices,^7^ boiling, heat transfer,^8^ and liquid–gas separation.^9^ By considering the emerging surface engineering techniques and bubble dynamics in two-phase flows, innovative methods for separating gas and liquid mixtures could be developed. The gas–liquid separators are one of the critical components of air-conditioning systems^10^ and compression systems.^11^ Improving the separator efficacy and decreasing the liquid residence time inside them can significantly affect the time and cost of the separation process.^12^

Furthermore, nanofibers and nanoporous materials can be utilized to resolve the problem of long-term floating micro- and nanobubbles within the liquid medium. The micro- and nanobubbles have a lower rising velocity in the liquid medium than macrobubbles, and since they have a lower buoyancy force, they can remain buoyant in the medium for an extended time period.^13^ Thus, developing a novel methodology to scavenge the floating micro- and nanobubbles within the medium and to merge them into larger bubbles can help in improving the efficiency of gas–liquid separators. The same approach can also be applied to other applications such as capturing methane gas bubbles from bodies of water. Methane gas bubble emissions from man-made and natural water bodies are among the sources of greenhouse gas emissions.^14^ Capturing the released methane gas bubbles before they enter the atmosphere could reduce their negative impact on the environment and global warming. Additionally, storing the harvested methane bubbles can be considered a potential energy source for power plants.

Water repellent surfaces can be classified as hydrophobic (water contact angle (WCA) > 90) and superhydrophobic (WCA > 150), depending on their WCA.^15^ Surfaces with a lower contact angle than 90° are considered hydrophilic. Hydrophobic and superhydrophobic materials repel the liquid phase to maintain contact between the solid surface and gaseous phase and to avoid formation of a new solid–liquid interface. Surface wettability can be altered by manipulating the chemical properties and surface energy^16^ and with the surface roughness and microstructures.^5,17^ Functionalizing the surface using nonpolar hydrophobic materials can improve the hydrophobicity by repelling the polar water molecules.^18^ The surface roughness elements, which can be created as precisely engineered surface porosities, are able to trap gas pockets and prevent liquid molecules from penetrating into the porosities.^19^ Using both of these approaches, it is possible to enhance the hydrophobicity of a surface. The higher projected surface area in the functionalized porous materials increases the surface density of the hydrophobic functional groups and boosts their effectiveness in repelling the liquid molecules. On superhydrophobic surfaces, the surface porosities can retain miniature gas bubbles and minimize the droplet’s contact with the surface in the Cassie–Baxter state. Under this condition, the surface tension of the penetrating liquid and the pressure of the gas bubble in the porosity play essential roles in preventing the liquid from leaking into the porosities.^20^ In the case of hydrophobic surfaces, the droplet can penetrate into the porosities in the Wenzel state.^21^

Electrospinning is an emerging fabrication technique for producing scalable, cost-effective, and easily controllable polymer nanofibers and membranes with hydrophobic and hydrophilic properties.^22^ The chemical properties of nanofibers can be tuned by selecting the appropriate polymers and their mixing ratio. It was reported in the literature that electrospun poly(methyl methacrylate) (PMMA) nanofibers have a high WCA and low water uptake.^23^ Our recent study confirmed that the WCA of electrospun PMMA nanofibers is about 124°, and the water droplets on the surface of the nanofiber are stable and do not penetrate into its structure.^24^ However, PMMA thin films and nonfibrous structures have been recognized as hydrophilic polymers with the WCA of 60–70°.^25^ The difference between the WCA of nanofiber and the thin film of PMMA can be attributed to the surface roughness and porosity. The air and water permeability of the membrane is greatly affected by the membrane’s hydrophobicity and hydrophilicity. Blending hydrophilic polymers such as poly(ethylene glycol) (PEG) with PMMA has been shown to significantly affect the hydrophobicity and swelling of electrospun nanofibers.^23,24^ Also, their physical properties, permeability, and surface roughness can be tailored to achieve the desired properties such as nanofiber diameter by optimizing the electrospinning parameters such as the accelerating voltage, spinneret to target distance, and humidity. Increasing the surface roughness on functionalized hydrophobic surfaces increases the projected surface area for mounting hydrophobic functional groups, which increases the stability of air packets within the porosities.^26^ Employing a dual strategy by engineering the chemical properties of the surface and its morphology leads to a higher WCA and more stable hydrophobicity.

There has been extensive research on the hydrophobicity and hydrophilicity of electrospun nanofibers in an air medium. However, their interaction with gas bubbles under submerged conditions has not been sufficiently explored. The interactions of the submerged hydrophobic and hydrophilic surfaces with gas bubbles can be estimated by studying the behavior of water droplets on their surface in the air medium. In addition to WCA, bubble contact angle (BCA) can also be useful for discerning the hydrophobic properties of submerged materials. The relationship between the BCA on a submerged surface and the WCA of the material in the air has been reported to be complementary on smooth surfaces;^27^ nevertheless, it was reported that this relationship could be violated on the surfaces with higher surface roughness and higher water contact angle hysteresis (CAH).^28,29^ The recent attempts to understand the interactions of hydrophobic and hydrophilic porous materials with liquid and gas bubbles revealed their potential in separating the gas phase from the two-phase media.^27,30^ Chen et al. fabricated a superhydrophobic material by modifying a sponge surface to study the methane bubble separation from water.^31^ They demonstrated that superantiwetting sponges can selectively absorb, store, and continuously transport marine methane bubbles. Ma et al. fabricated superhydrophobic three-dimensional (3D) gradient porous interconnected network surfaces to study the possibility of macro- and microgas bubble transportation in a liquid medium.^32^ The prepared and treated copper wires in that study could maintain a constant gas film underwater for an extended period of time and transported the gas film using the Laplace pressure difference. Chen et al. investigated the air bubble interactions with Janus wettability membranes, which had an asymmetric surface wettability. They showed that while the air bubbles on the hydrophilic side of the membrane tended to penetrate upward into the mesh, the air bubbles on the superhydrophobic side favored horizontal spreading on the surface of the fabricated air “diode”.^33^ Although there are some studies on the interactions of hydrophobic and hydrophilic surfaces with air bubbles, the mechanism of air bubble capture, its penetration behavior, and transportation through electrospun nanofibers are yet to be explored. Therefore, revealing the mechanism of the trapped bubbles on nanofibers and understanding their interactions are of utmost importance, particularly for the researchers and engineers working on bubble dynamics, capture, separation, and transportation.

In this study, hydrophobic and hydrophilic electrospun nanofibers of PMMA and PMMA–PEG were fabricated to investigate the gas bubble interactions on submerged nanofiber mats. PMMA nanofibers were selected as hydrophobic polymers, and the PEG polymer was added to the PMMA polymer for tuning the hydrophobicity of PMMA–PEG nanofibers. The WCA and underwater BCA of the electrospun nanofibers were measured. The behavior of gas bubbles, including bouncing and attachment and penetration in the vicinity of the nanofibers, was extensively examined using a high-speed camera, and the gas-phase diffusion mechanism was explored in detail. The prepared nanofibers were reinforced by a plastic mesh to improve their durability. The mechanical properties of the electrospun nanofibers and reinforced nanofibers were studied via a tensile test stand. The gas permeability of the electrospun and reinforced nanofibers was also visualized using a homemade setup to investigate the effect of electrospun nanofibers on the bubble coalescence and size in addition to the frequency of released bubbles from the nanofiber mat. The reported results showed that the presented novel approach could be used to separate the gas phase from the liquid by sorting and reducing the size distribution of gas bubbles.

## Materials and Methods

### Materials

*N*,*N*-Dimethylformamide (≥99%), PMMA (*M*_w_ = 3 50 000 g/mol), and PEG (average *M*_n_ ∼ 400) were purchased from Sigma-Aldrich (technical grade) and were used without any purification. A Milli-Q device was used to obtain deionized water (resistance 18.2 MΩ cm).

### Electrospinning of Hydrophobic and Hydrophilic Nanofibers

The electrospinning of nanofibers was carried out based on our research group’s recent study, where optimization of the electrospinning parameters and characterization of the physical and chemical properties of electrospun nanofibers were reported.^24^ Based on the reported properties, the PMMA and PMMA-30%PEG nanofibers were electrospun using a 12 kV electric field, spinneret and collector distance of 20 cm, and flowrate of 0.6 mL h^–1^.

### Mechanical Characterization of Electrospun Nanofibers

A plastic mesh with 2 mm × 2 mm apertures was used as a support to improve the mechanical properties of the membrane. The mechanical properties of electrospun nanofibers and reinforced mat with the plastic mesh were characterized using a Mark-10 ESM 303 motorized tensile test stand and M7–020 digital force gauge based on the ASTM D882-10 standard test method for tensile properties of thin plastic sheeting. The surface of electrospun nanofibers was studied using the field emission scanning electron microscopy technique (FESEM, LEO Supra VP-55).

### Visualization of the Water and Gas-Phase Interactions with Electrospun Nanofibers

**Figure 1:**
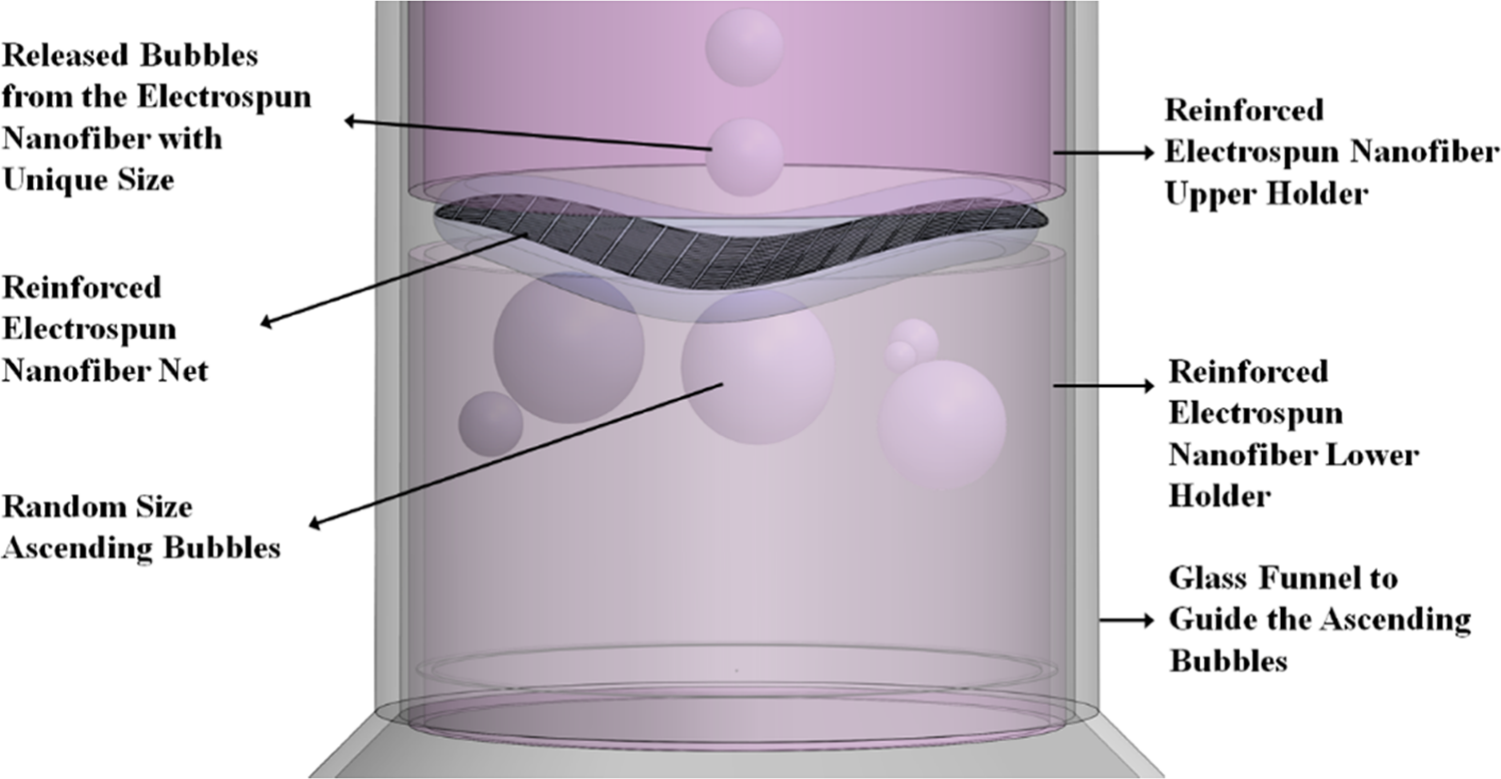
Assembly of the reinforced electrospun nanofiber in the funnel.

WCA measurements were conducted using the Attension Theta Lite tensiometer. After accumulating a 5 μL droplet of distilled water (18.2 MΩ mm at 25 °C) on the tip of the needle, the droplet was released by moving the syringe downward and contacting the sample surface. The BCA on submerged electrospun nanofibers and the diffusion mechanism of air bubbles through electrospun nanofibers were investigated by assembling the reinforced nanofibers on a glass funnel, as shown in Figure 1. The setup was visualized using a CMOS double-shutter camera (VEO-710), and a cold light halogen source was used to attain appropriate lighting for different imaging rates. A set of tube holders held the reinforced nanofibers in place, while the funnel directed rising air bubbles toward the nanofiber mat.

## Results and Discussion

In a recent study of the authors,^24^ 20, 30, and 40% PMMA–PEG solutions were prepared, and the properties and uniformity of the electrospun nanofibers were investigated. Although the addition of different amounts of PEG to the PMMA polymer could have a significant impact on its wettability properties, its impact on the electrospinning conditions and quality of the prepared nanofibers should not be underestimated. This study showed that a lower amount of the PEG polymer could not have any noticeable effect on the wettability properties of nanofibers. A higher amount of PEG decreases the uniformity of the electrospun nanofibers and causes the generation of beads on the surface of the mat. These beads not only reduce the topographical uniformity of the surface but also have a negative impact on the uniformity of the nanofiber chemical composition. Taking all of the abovementioned effects into consideration, the PMMA-30%PEG was selected in this study as an optimum mixing ratio to exploit the best properties from the prepared electrospun nanofibers.

**Figure 2:**
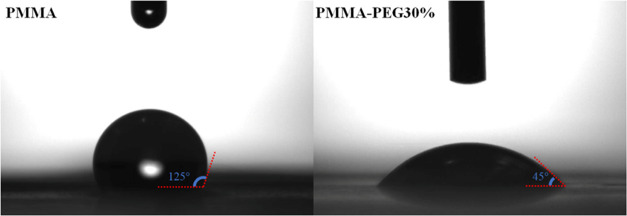
Water contact angles of PMMA and PMMA-30%PEG nanofibers.

Figure 2 shows the WCAs on PMMA and PMMA-30%PEG nanofibers. The WCAs for PMMA and PMMA-30%PEG were measured as 125 and 45°, respectively. PEG is a hydrophilic polymer and dissolves in water when it comes to contact with water droplets.^34^ The oxygen atoms on ether groups in the backbone of PEG absorb hydrogen atoms, resulting in a high solubility of PEG in water.^35^ The WCA of the thin film of PMMA was reported as 65° in the literature;^25^ however, this value can reach higher values such as 160° in fibrous mats.^23^ This behavior can be attributed to the effect of surface roughness on hydrophobicity. Blending these two polymers leads to hydrophobic and hydrophilic regions on the surface of electrospun nanofibers and reduces the WCA of PMMA-30%PEG in comparison with PMMA. Meanwhile, PMMA reduces water molecules’ accessibility to PEG and prevents their dissolution. Therefore, despite the high water solubility of PEG, PMMA-30%PEG is stable in water.^23^

The water stability of the electrospun nanofibers was characterized by measuring the weight changes of the submerged electrospun nanofibers in different time intervals. Samples (25 cm^2^) of fresh electrospun nanofibers were placed in a ventilated oven at 40 °C for 4 h and then weighed using a four-digit analytical balance. The dried nanofiber was submerged in water for 1, 2, 5, 10, 24, and 72 h and then dried again in the oven for 4 h to get rid of the infused water into the structure of the electrospun nanofibers. The weight changes of the nanofibers were measured with a precision of 0.1 mg, and almost no weight difference was measured during the experiments. Therefore, it can be concluded that PMMA-30%PEG is completely stable in an aqueous environment.

**Figure 3:**
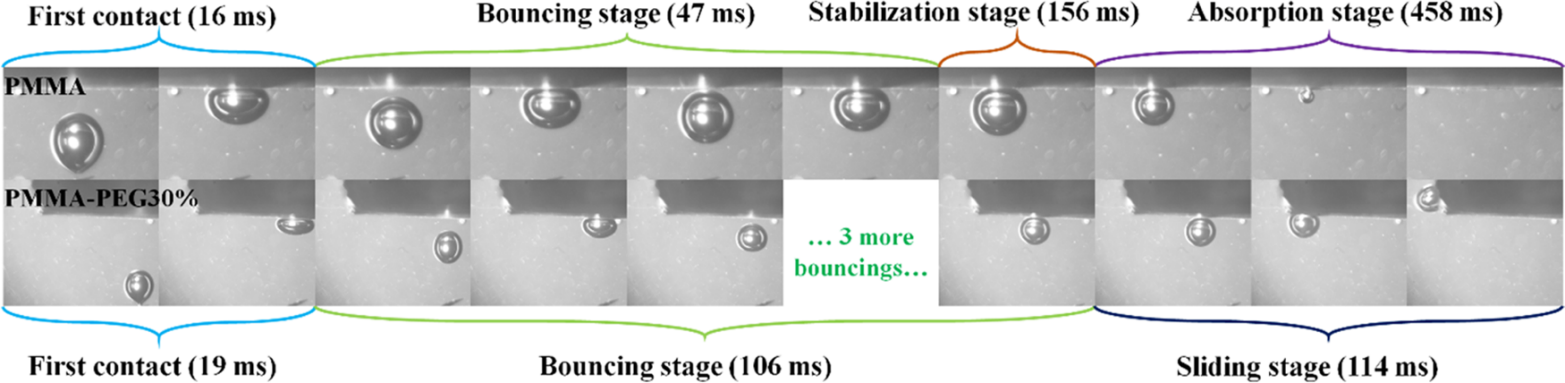
Bubble bouncing, absorption, and sliding on the surface of PMMA and PMMA-30%PEG electrospun nanofibers.

Figure 3 shows the air bubble interaction with submerged electrospun nanofibers. The air bubble volume, which was generated using a 0.4 mm needle, was 6 μL. The speed of the air bubble reaching the surface of the nanofiber mat was 0.16 m/s. Although the WCA measurements of the PMMA mat suggest that the electrospun mat has hydrophobic properties, BCA does not exhibit any aerophilic behavior and is about 160°. The air bubble does not spread on the surface of the PMMA mat, and it bounces off the surface a few times to dissipate its kinetic energy and rupture the water layer on the surface. The collision between the air bubble and surface is controlled by the van der Waals forces between the water molecules and membrane surface. The surface’s hydrophobic properties reduce the effect of the van der Waals forces and facilitate the bubble absorption on the surface.^27^

Consequently, the bouncing of the air bubble on the surface of PMMA-30%PEG nanofibers takes place 2–3 times more than that of PMMA nanofibers. The air bubbles reach the surface of hydrophobic and hydrophilic electrospun nanofibers simultaneously, and the speed of the impact is identical. However, the bouncing stage takes 47 and 106 ms long for hydrophobic PMMA and hydrophilic PMMA-30%PEG nanofibers, respectively. The time difference in stabilizing the air bubble on the surface reveals the difference in the van der Waals interactions of the water molecules and the surface of each nanofiber. The thin water layer between the hydrophilic nanofiber and bubble is more stable than that of hydrophobic nanofibers due to the van der Waals forces between water and hydrophilic nanofiber molecules. Therefore, it becomes more difficult for the gas bubble to overcome the force and to rupture the thin water layer to reach the surface of the nanofiber.

The nanofibers were assembled on a horizontal holder for these measurements. The gas bubble on the PMMA nanofiber diffuses into the porosities of the nanofiber after the bouncing stage within 458 ms; however, the air bubble on PMMA-30%PEG nanofibers slides on the surface with a bubble contact angle hysteresis (BCAH) close to zero as shown in the absorption stage and sliding stage in Figure 3, respectively. Considering the air bubble volume and its occupied area on the nanofiber, the diffusion rate of air bubbles in PMMA electrospun nanofibers is 10 L/s for each square meter of the nanofiber.

**Figure 4:**
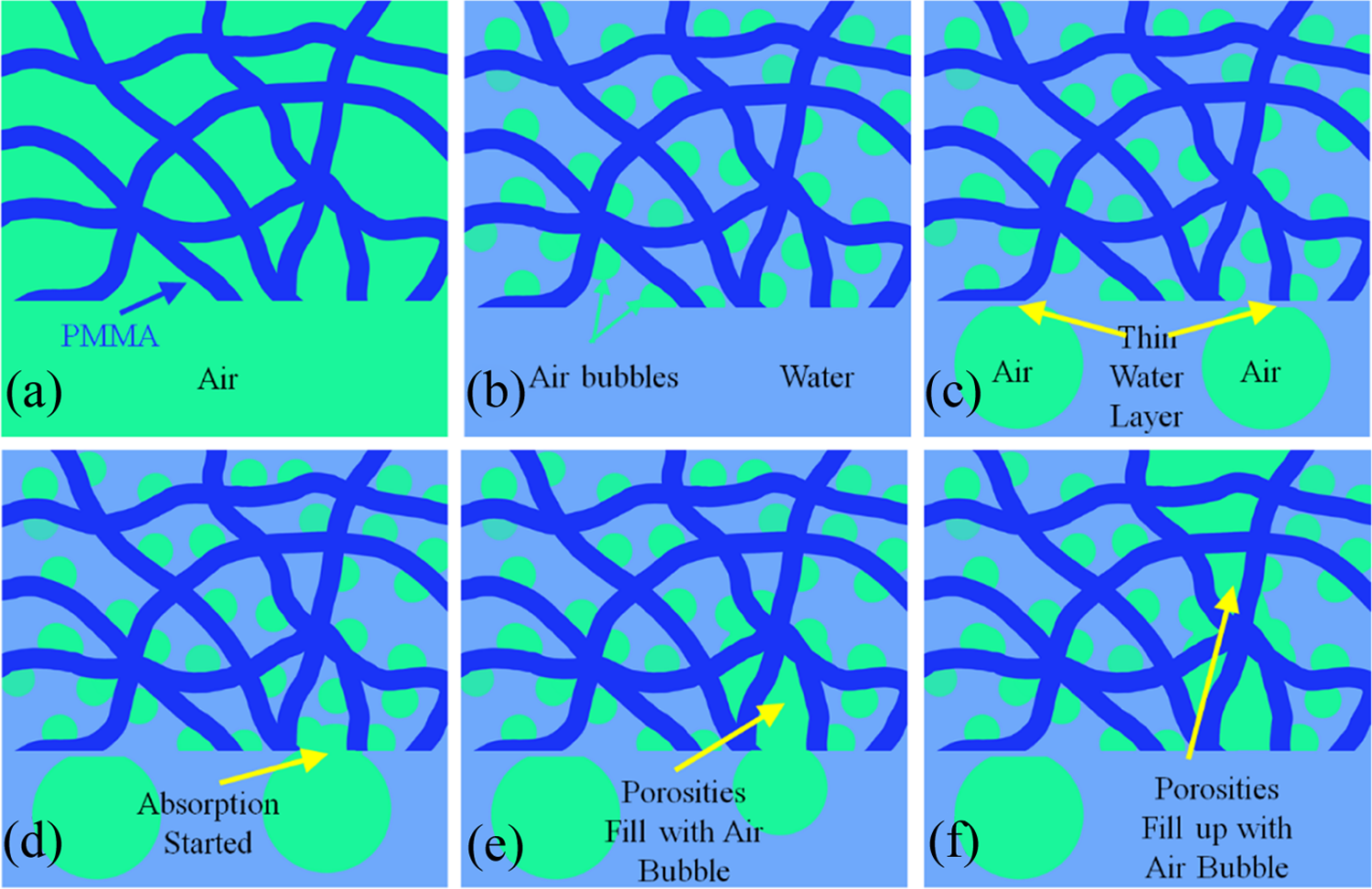
(a–f) Schematic presentation of PMMA electrospun nanofiber’s sequential interaction with air bubble and water.

Figure 4 represents the schematic presentation of a single air bubble’s interaction with submerged PMMA electrospun nanofibers. The porosities of the dry PMMA electrospun nanofibers before submerging in water are filled with air (Figure 4a). The water molecules penetrate into the porosities by submerging the nanofibers in water. Depending on the compactness of nanofibers and their distance from each other, trapped air is expelled from the fiber net. However, the regions close to nodes or overlapping nanofibers retain the air bubble (Figure 4b). Nanofibers in these regions act together to increase the strength of the hydrophobic force, thereby repelling the water molecules more effectively. When a gas bubble rises toward the nanofibers, a thin layer of water prevents the contact between the approaching gas bubble and nanofibers (Figure 4c). However, due to the hydrophobicity of nanofibers, which is responsible for the presence of miniature trapped gas bubbles on the surface of the nanofibers, approaching gas bubbles coalesce with the trapped miniature gas bubbles on the surface and find a path to penetrate into the porosities and deflate into the nanofiber net (Figure 4d,e). However, some of the approaching gas bubbles cannot overcome the van der Waals forces between the nanofiber and water layer and slide over the surface with low BCAH (Figure 4f).

**Figure 5:**
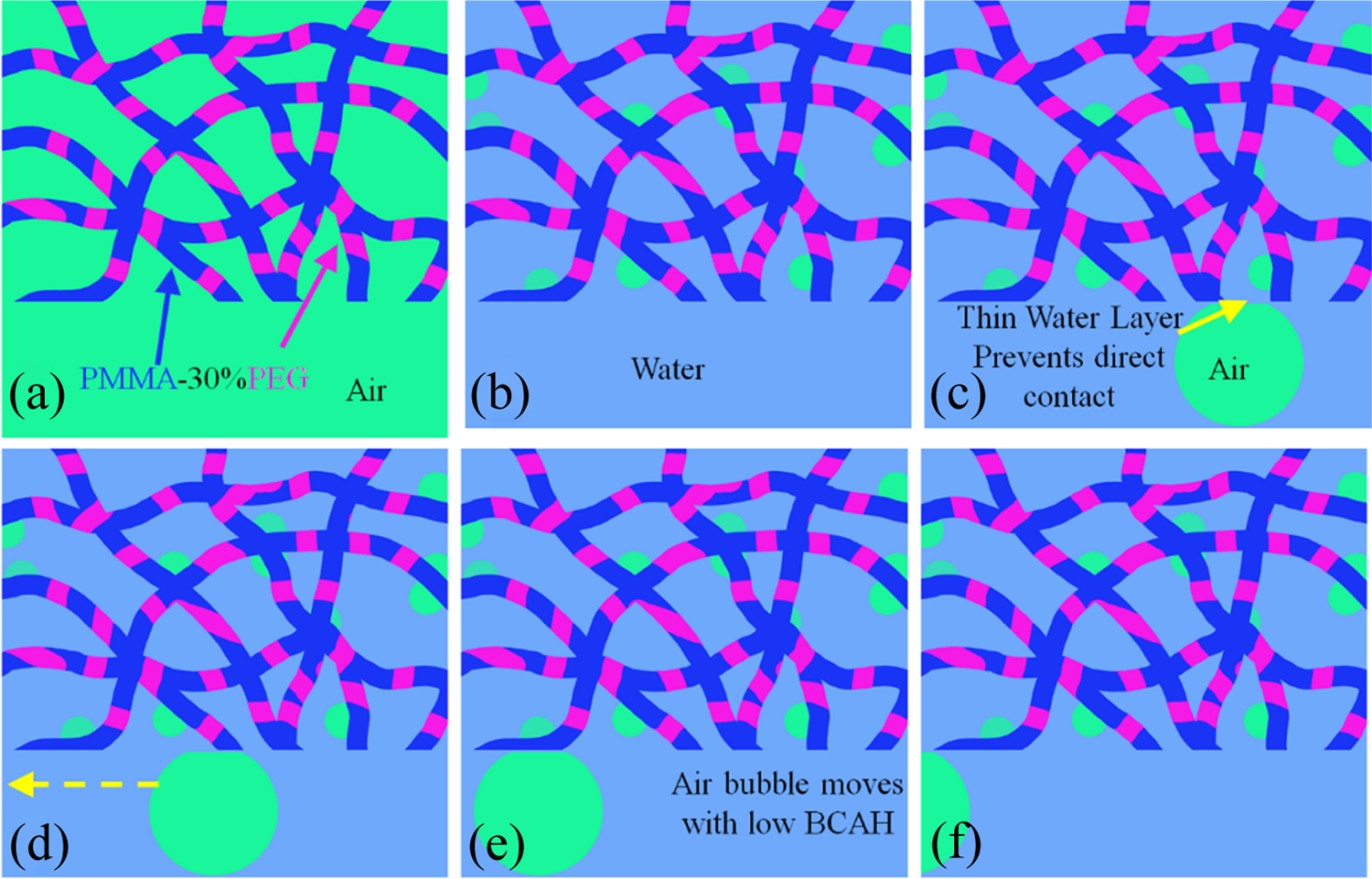
(a–f) Schematic presentation of PMMA-30%PEG electrospun nanofiber’s sequential interaction with air bubble and water.

Figure 5 shows the interactions of electrospun hydrophilic PMMA-30%PEG nanofibers with gas bubbles. PMMA-30%PEG electrospun nanofibers are less effective at trapping miniature air bubbles than PMMA electrospun nanofibers due to the presence of hydrophilic PEG polymers (Figure 5a). The water molecules can form a hydrogen-bond network with PEG polymers similar to bulk water because of the oxygen atoms’ order on the polymer’s backbone. Therefore, adding PEG to the PMMA polymer reduces the WCA and hydrophobicity of the electrospun nanofiber.^35^ Meanwhile, the attraction forces between the electrospun nanofiber and the approaching gas bubble are insufficient to overcome the van der Waals forces between the water layer and nanofibers. Therefore, the air bubble cannot penetrate into the porosities of the hydrophilic nanofiber net. Furthermore, sliding off the air bubble on the surface of the electrospun nanofiber with a BCAH of close to zero confirms the presence of the thin water layer.

**Figure 6:**
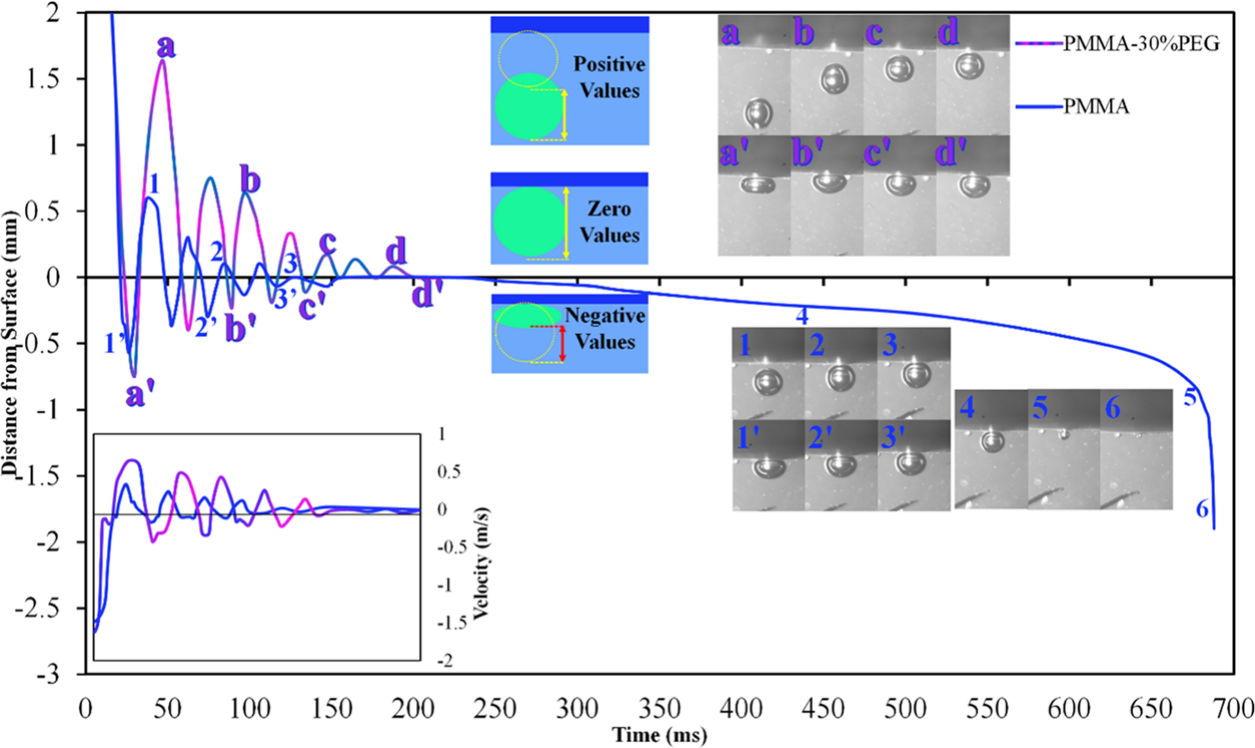
Bouncing and absorption behavior of the air bubble on the surface PMMA and PMMA-30%PEG electrospun nanofibers with time.

Figure 6 shows the bouncing sequences of the approaching gas bubble to electrospun PMMA and PMMA-30%PEG nanofibers. This figure shows the changes in the distance between the farthest point on the gas bubble and the nanofiber surface over time. This value for a resting bubble on the surface, which is equal to its diameter, is considered zero. While the positive values indicate the gas bubble’s bouncing, the negative values represent the bubble’s compression due to the impact. Although the velocity and volume of approaching gas bubbles to PMMA and PMMA-30%PEG electrospun nanofibers were kept constant during the experiments, the impacting gas bubble on the hydrophilic PMMA-30%PEG nanofiber bounces 3 times more than hydrophobic PMMA nanofibers. Furthermore, the bouncing distance of gas bubbles on PMMA-30%PEG nanofibers is 3 times larger than hydrophobic PMMA nanofibers. The gas bubble impacting hydrophilic nanofibers bounces away farther than the gas bubble impacting hydrophobic nanofibers and bumps into the surface with greater kinetic energy in the next cycle. However, the compression of the gas bubbles on hydrophobic nanofibers is more significant than that on hydrophilic nanofibers. As illustrated in Figure 6, miniature bubbles are retained in the porosities of hydrophobic nanofibers, which facilitate the attachment of the rising bubbles to the membrane surface by dissipating their kinetic energy.^36^ The hydrophobic properties of PMMA cause the approaching gas bubble to dissipate more energy and bounce less than the gas bubble on hydrophilic PMMA-30%PEG.

Interestingly, the bouncing bubble on the surface of PMMA-30%PEG reaches the terminal velocity for a complete detachment from the surface of the membrane (Figure 6a–d); however, the rising bubble toward the hydrophilic nanofiber does not leave the surface of the membrane after the first collision (Figure 6). The velocity changes of approaching bubbles toward PMMA-30%PEG and PMMA membranes show that the rising bubbles for both membranes have almost the same initial velocity. The bouncing bubble on the hydrophobic nanofiber cannot be detached from the surface of the membrane after the first impact; however, it can be detached from the hydrophilic membrane. Therefore, the terminal velocity for detachment of the gas bubble from the membrane is greater than 0.33 m/s and less than 0.64 m/s. Furthermore, the gas bubble deceleration rate is much higher on the hydrophobic surface than on the hydrophilic surface since the gas bubble velocity diagram slope is steeper in the hydrophobic membrane’s diagram.

**Figure 7:**
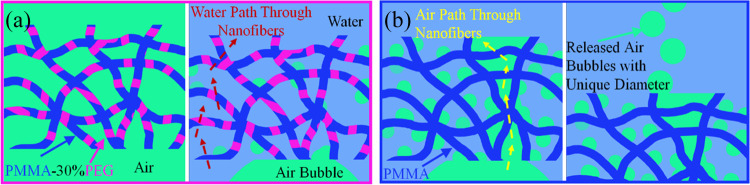
Water and air diffusion in the (a) hydrophilic PMMA-30%PEG electrospun nanofibers and (b) hydrophobic PMMA electrospun nanofibers.

After stabilization of the gas bubble on the membranes’ surface, the bubble on hydrophilic nanofibers does not diffuse into its porosities. However, the hydrophobic nanofiber absorbs the bubble within about 400 ms. The air and water diffusion mechanisms in PMMA and PMMA-30%PEG electrospun nanofibers are illustrated in Figure 7. The submerged nanofibers retain miniature gas bubbles in their structure; however, due to the hydrophobic properties of PMMA nanofibers, the density and stability of trapped air bubbles in the structure are higher in comparison with hydrophilic PMMA-30%PEG nanofibers. Therefore, by increasing the number of approaching gas bubbles and their penetration into the structure of the nanofiber, they can easily be substituted with water inside the nanofiber. On the other hand, hydrophilic nanofiber mats can provide suitable paths for the diffusion of water molecules and prevent the passage of air molecules.

**Figure 8:**
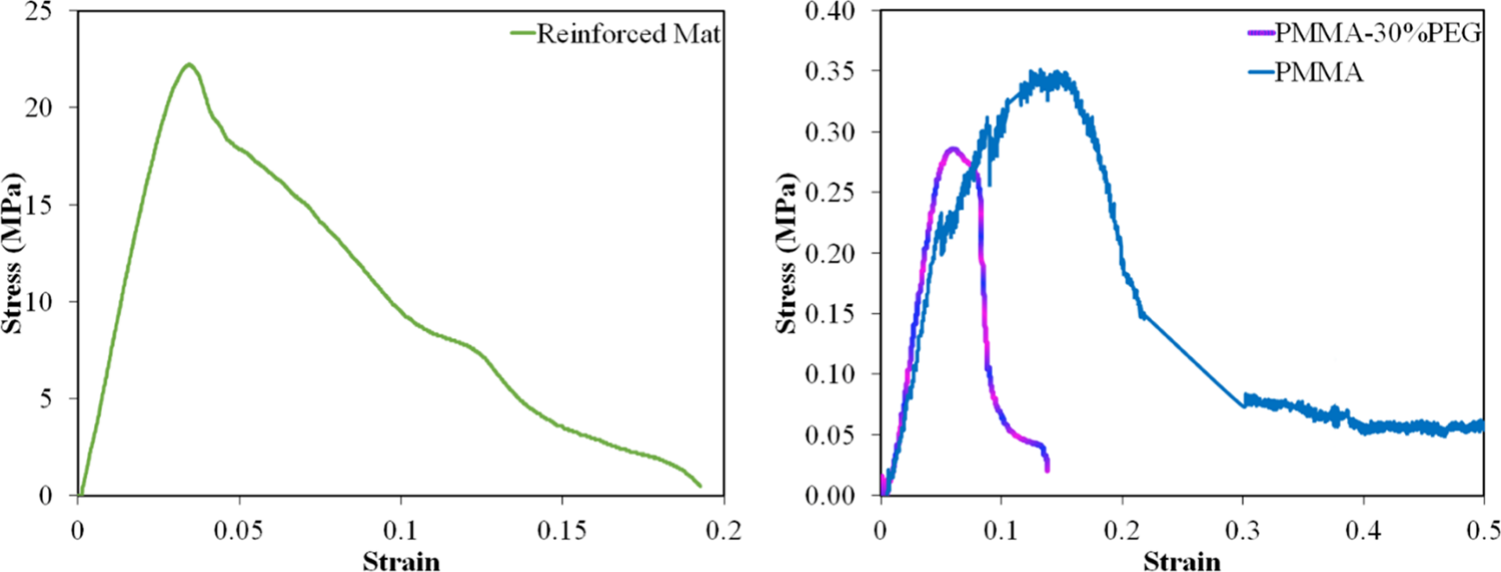
Mechanical properties of PMMA-30%PEG electrospun nanofibers and the reinforced nanofiber mat.

The stress–strain curve of PMMA and PMMA-30%PEG electrospun nanofibers and supporting plastic mesh is displayed in Figure 8. The ultimate tensile strengths of the PMMA and PMMA-30%PEG nanofibers were measured as 0.35 and 0.30 MPa, respectively. The applied tension force on the nanofibers realigns their orientation in the direction of the force. The fluctuations in the stress–strain curves of PMMA electrospun nanofibers and smoothness of PMMA-30%PEG nanofibers can be related to the compactness of PMMA-30%PEG in comparison with PMMA nanofibers. Although a rise in the electrospinning time can increase the thickness of the nanofiber mat and improve its mechanical strength, the penetration and diffusion of gas bubbles through nanofibers can be negatively affected.

**Figure 9:**
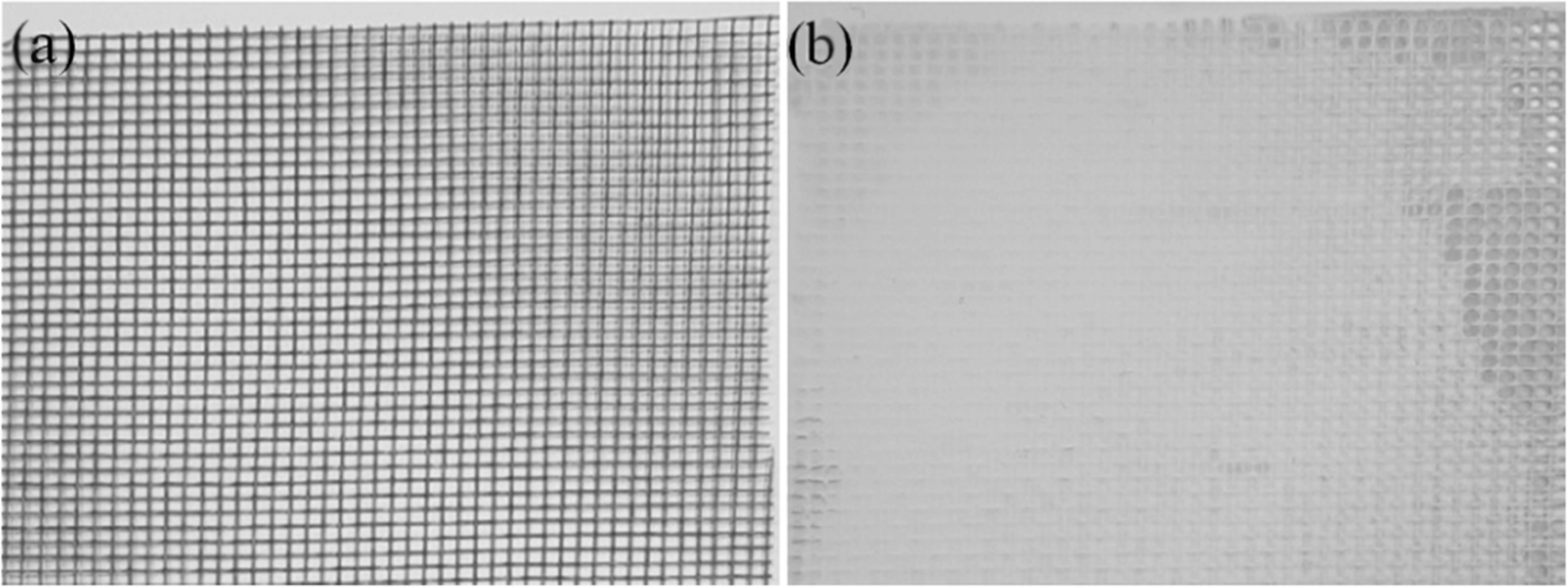
Plastic mesh with an aperture size of 2 mm × 2 mm (a) before and (b) after electrospinning of the nanofiber mat.

Furthermore, the consumed materials required for electrospinning the nanofiber mat and their costs can also increase. In this regard, a cheap and mechanically durable plastic mesh was used to improve the mechanical properties of the membrane without having a negative impact on its performance. Supporting the electrospun nanofibers using the plastic mesh increases the mechanical properties of the final membrane by a factor of 70 (Figure 8). The plastic mesh before and after electrospinning of the nanofibers on its surface is shown in Figure 9. As can be seen, the electrospun nanofiber mat on the plastic mesh has a uniform surface, and the presence of the plastic mesh does not adversely affect the quality of the mat.

**Figure 10:**
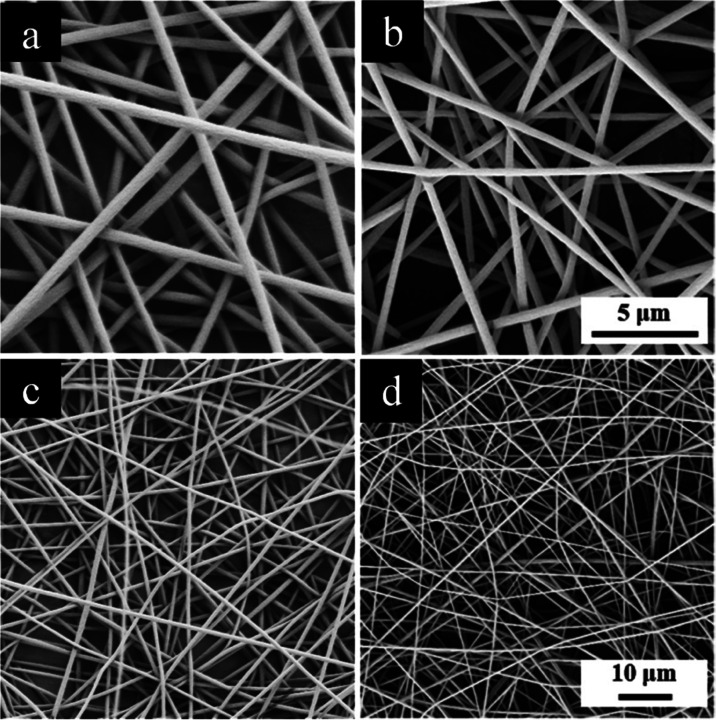
SEM images of (a, c) PMMA and (b, d) PMMA-30%PEG nanofibers.

SEM images of electrospun PMMA and PMMA-30%PEG nanofibers are shown in Figure 10. The average diameters of nanofibers measured using the ImageJ software are 600 and 450 nm, respectively. Moreover, no beads were observed on the nanofiber mats, and their surface was uniform and smooth. The electrospinning parameters, solution concentration, and solvent for each sample are unique; however, upon the addition of the PEG polymer to the electrospinning solution, the diameter of nanofibers decreases. The conductivity of PMMA and PEG polymers was reported in the literature.^37^ The addition of the PEG polymer to the solution increases the conductivity of the polymer blend, thereby leading to an amplification in the effect of the electrical field on the electrospinning jet. The electrospun nanofibers are subjected to a higher force and stretch during the electrospinning process. Therefore, the diameter of the nanofibers decreases upon addition of the PEG polymer.^38^

**Figure 11:**
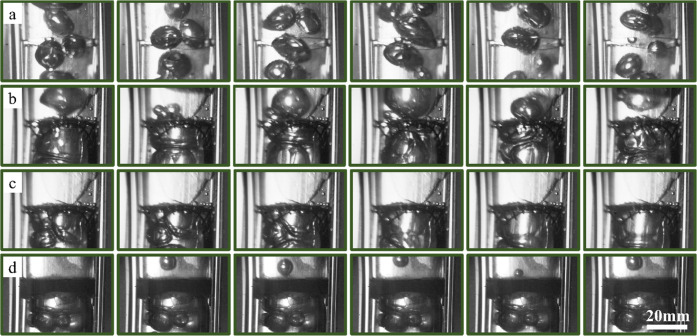
Bubble ascending in the funnel. (a) Without membrane and plastic mesh. (b) With plastic mesh. (c) Reinforced electrospun PMMA-30%PEG. (d) Reinforced electrospun PMMA (the time interval is 12 ms). The hydrophobic reinforced electrospun nanofibers pass the gas bubbles with a specific volume and frequency; however, the hydrophobic reinforced nanofibers do not allow the passage of the gas bubbles due to the stability of the water molecules in the structure of the electrospun nanofibers.

Figure 11 shows the interaction of the plastic mesh and reinforced electrospun nanofibers with ascending bubbles, as illustrated in Figure 1. In Figure 11a, the bubbles with a large size distribution pass through the neck of the funnel, which is not equipped with a membrane. While ascending, neither the bubbles coalesce, nor their sizes change significantly. Figure 11b shows the funnel with the plastic mesh that prevents bubbles from free ascending and makes them coalesce before passing through the mesh. Each passing air bubble has a different diameter, and the frequency of its release is random. The reinforced hydrophilic PMMA-30%PEG nanofiber completely blocks the passage of air bubbles (Figure 11c). The stored air bubbles behind the membrane coalesce and create an air column without leakage. As illustrated in Figure 5, this behavior can be explained by the continuous water layer retained in the structure of hydrophilic electrospun nanofibers. In the case of reinforced PMMA nanofibers (Figure 11d), the rising bubbles of varying diameters coalesce and create an air reservoir behind the membrane. The miniature air bubbles in the pores of the membrane facilitate air diffusion into the membrane and provide a passage to the surface for gas bubbles. From the upper part of the membrane, gas bubbles are released at a predictable frequency and have a defined diameter. The diameter of bubbles introduced into the membrane ranges from 2 to 25 mm; however, the diameter of bubbles released from the hydrophobic electrospun nanofibers is 8 ± 1 mm. It has been reported in the literature that the hydrophobicity and hydrophilicity of electrospun nanofibers have a significant impact on the behavior of the interacting solid, liquid, and gas phases in aqueous solutions on the surface of the mat.^13,28,39^ These properties can be adjusted by engineering the chemical composition of the nanofibers and their electrospinning parameters.^40^ This study proves that it is possible to capture a wide size distribution of air bubbles using electrospun nanofibers and to release them with a constant diameter. These observations are consistent with the mechanism displayed in Figure 7b. Further investigations and optimization efforts are needed to tailor the released bubble diameter depending on the targeted applications.

## Conclusions

In this study, the interaction of gas bubbles on submerged nanofiber mats made of hydrophobic and hydrophilic electrospun nanofibers, which were fabricated from PMMA and PMMA–PEG polymers, was investigated. The WCA measurements showed that PMMA and PMMA-30%PEG nanofibers have WCAs of 125 and 45°, respectively. The oxygen atoms on ether groups in the backbone of PEG absorb hydrogen atoms in water, resulting in a high solubility of PEG; however, mixing it with PMMA reduces the water molecules’ accessibility to PEG and prevents their dissolution. Therefore, PMMA-30%PEG becomes a hydrophilic and undissolvable polymer in water. High-speed visualization of the rising gas bubbles revealed that they have no tendency to spread on the surface of the PMMA mat; they bounce off a few times to dissipate their kinetic energy and rupture the thin layer of water on the surface. The bouncing stage takes 47 and 106 ms long for hydrophobic PMMA and hydrophilic PMMA-30%PEG nanofibers, respectively. The time difference in stabilizing the air bubble on the surface suggests the difference in the van der Waals interactions of the water molecules and the surface of each nanofiber. The hydrophobic properties of the surface reduce the effect of the van der Waals forces and facilitate the bubble absorption on the surface. Considering the air bubble volume and its occupied area on the nanofiber, the diffusion rate of air bubbles in PMMA electrospun nanofibers is 10 L/s for each square meter of the nanofiber.

Furthermore, sliding off the air bubble on the surface of the hydrophilic electrospun nanofiber with a BCAH of close to zero confirms the presence of the thin water layer on the surface. The bouncing bubble on the hydrophobic nanofiber cannot be detached from the surface of the membrane after the first impact; however, it can be detached from the hydrophilic membrane. Therefore, the terminal velocity for detachment of the gas bubble from the membrane is greater than 0.33 m/s and less than 0.64 m/s. The ultimate tensile strengths of the PMMA and PMMA-30%PEG nanofibers were measured as 0.35 and 0.30 MPa, respectively, and the reinforced nanofibers have 70 times more strength. The conductivity of the solution in PMMA–PEG blend was higher than the PMMA electrospinning solution. Therefore, the PMMA–PEG electrospinning jet was stretched more under the same electric field, and the diameter of the electrospun nanofibers decreased in comparison with PMMA nanofibers. Finally, the diameter of the bubbles introduced into the membrane ranges from 2 to 25 mm, while the diameter of the bubbles released from the hydrophobic electrospun nanofibers is 8 ± 1 mm. These results show that the reinforced hydrophobic electrospun PMMA–PEG nanofibers have a high potential in capturing, sorting, and releasing the gas bubbles with a predictable frequency and size distribution.
